# Nutritional Status of Patients Diagnosed with Cervical Cancer in Tanzania

**DOI:** 10.21203/rs.3.rs-7780798/v1

**Published:** 2025-12-18

**Authors:** Li Zhang, Chacha J. Mwita, Ghada A. Soliman, Dativa F. Mushi, Germana Leyna, Amr S. Soliman, Esther M. Nkuba

**Affiliations:** City University of New York School of Medicine; Ocean Road Cancer Institute; City University of New York School of Public Health; Ocean Road Cancer Institute; Tanzania Food and Nutrition Center; City University of New York School of Medicine; Tanzania Food and Nutrition Center

**Keywords:** ORCI, Tanzania, Cervical Cancer, HPV, Nutrition, PG-SGA

## Abstract

**Background:**

Cervical cancer is the most common cancer affecting Tanzanian women. The nutritional status of patients with cervical cancer may influence cervical cancer outcomes. However, no information is available regarding the nutritional status of the Tanzanian cervical cancer patients. Therefore, we evaluated the nutritional status of patients with cervical cancer treated at the Ocean Road Cancer Institute (ORCI) in Tanzania.

**Methods:**

We conducted a cross-sectional study of 184 newly diagnosed patients with cervical cancer seen at the oncology clinic of ORCI from April to September 2023. Assessment of the nutritional status was based on the Patient-Generated Subjective Global Assessment (PG-SGA) scores. The overall nutritional status was evaluated using nutritional blood biomarkers, food intake frequencies, a categorical rating indicating the level of malnourishment, and a triage recommendation. Multivariate regression analysis was performed to predict nutritional status based on demographic and clinical variables.

**Results:**

Among the total patient population, 39.1% reported consuming less food after diagnosis, especially the intake of cereals, dark leafy green vegetables, orange vegetables, other vegetables, as well as oils, and fats. The majority (84.8%) of patients required nutritional intervention based on their nutritional status. Almost half (48%) of patients with early-stage cervical cancer (stages I, II) and 78% of patients with late-stage cervical cancer (Stage III, IV), showed moderate or severe malnutrition. The risk of malnutrition was significantly higher in patients with late-stage vs early-stage cancer (8.0 ± 1.4 vs. 5.9 ± 0.8; *p* = 0.0173). Similarly, patients with late-stages had higher mean levels of white blood cells (late vs. early: 9.0 ± 1.1 vs. 7.6 ± 0.6; *p* = 0.0216), platelets (396.3 ± 39.4 vs. 347.5 ± 23.9; *p* = 0.0399), the ratio of platelet to neutrophil count (101.7 ± 13.8 vs. 82.3 ± 1.6; *p* = 0.0294), and the liver function test, aspartate aminotransferase (AST) enzyme (31.9 ± 5.9 vs. 24.5 ± 2.0; *p* = 0.0201). Late-stage patients were predicted to have a worse nutritional status (late vs. early: mean = 7.8 vs. 6.0, p = 0.04), after adjusting for sociodemographic covariates. Further adjustments by smoking, alcohol use and physical activity did not affect the model.

**Conclusion:**

Nutritional status of patients with late-stage cervical cancer in Tanzania should be prioritized during the management of patients with cervical cancer, especially for late-stage patients. This study may have translation to other low-income countries facing challenges in cancer management, poverty, and challenges in food security.

## Introduction

Cervical cancer is a significant and most common cancer affecting women in Sub-Saharan Africa, including Tanzania [1–3]. Cancer mortality among patients with cervical cancer in Tanzania is high (age-standardized incidence rate = 42.2/100,000), and late-stage presentation, as well as short survival among Tanzanian patients with cervical cancer, call for interventions for improving patient management [4].

Tanzania has faced a high cervical cancer incidence (age-standardized mortality rate = 64.8/100,000) and malnourishment [2, 4–6]. Cervical cancer is caused by the human papillomavirus (HPV) [7], and past research showed that the nutritional status of patients may modify cervical cancer outcomes and response to treatment [8–10].

Patients with cancer and poor nutritional status may suffer from neutropenia quickly and, therefore, may not be able to tolerate chemotherapy and radiotherapy treatment. Neutropenia is common among patients with cancer in Tanzania [11, 12]. However, information regarding the nutritional status of patients with cervical cancer in Tanzania, which may be associated with lower white blood count and neutropenia, is lacking. Therefore, we conducted this study to characterize the nutritional status of patients with cervical cancer treated at the Ocean Road Cancer Institute (ORCI) in Dar es Salaam, Tanzania.

## Methods

### Study Location

This cross-sectional study was conducted at the ORCI, Tanzania’s largest and oldest cancer center. ORCI sees patients from all over Tanzania with diverse socioeconomic, geographic places of residence and demographic backgrounds. Patients seeking care at the ORCI also represent a wide range of disease stages, as ORCI offers chemotherapy and is one of the very few radiotherapy programs in the country.

### Study Population and Data Collection

The study included all newly diagnosed patients with cervical cancer seen at the oncology clinic of ORCI during the period from April to September 2023. Patients with cervical cancer seen at ORCI represent the general patient population of Tanzania. Based on Tanzania’s latest census of 2022 [13] and our previous publications on cervical cancer at ORCI ([14, 15] and Fuss et al., 2024), the most populous regions of Tanzania (Dar es Salaam, Mwanza, Tabora, Morogoro, and Dadoma) as well as the less populated regions of Tanzania are represented in the patient population of ORCI.

All 184 patients who were approached for the study agreed to participate. All interviews were conducted at the beginning of the patients’ appointments, before any discussion by the medical team about diagnosis or treatment. Every patient included in the study provided verbal consent to participate, as documented in the ORCI IRB approval. The patients’ mean age was 53.3 ± 11.1. Data collection was conducted through all the following three approaches: A) conducting dietary interviews of all newly diagnosed patients with cervical cancer who were seen at the oncology clinic of ORCI during the period from April to September 2023. Patients were asked about their dietary intake on a typical day before diagnosis and within 24 hours after diagnosis. The interviews used a Tanzania Food Frequency Questionnaire (FFQ). The questionnaires were administered by an experienced local nutritionist and a co-author (DFM). B) Clinical and demographic data of the patients interviewed were retrieved from their medical records, and C) existing laboratory biomarker data were abstracted from the medical records of all interviewed patients. Blood biomarker analysis is routinely performed on-site, after the initial clinical examination of all patients, and before any treatment is prescribed at ORCI. The study was approved by the ORCI Research Ethics Committee. The IRB approval of ORCI was done according to the Declaration of Helsinki. The City University of New York (CUNY) determined that this study is exempt from the Institutional Review Board (IRB) review because the work done at CUNY included only statistical analysis of de-identified datasets.

## Assessment of the Nutritional Status

The nutrition assessment score (1–24) was derived from the validated Patient-Generated Subjective Global Assessment (PG-SGA) with modifications [13]. The PG-SGA questionnaire was in English, and the native speaking interviewer from ORCI translated the questions to the patients and translated their responses back into the questionnaires. The scores were calculated based on the nutritional and health indicators, including changes in food intake, weight change, symptoms, comorbidities, disease stage and its relation to nutritional requirements, activities, metabolic demands, and physical examination (loss of muscle mass or body fat, edema or ascites) that were captured in the clinical nutritional assessment form. The respective indicator included a change in weight change (0–1), food intake (0–4), symptoms (0–3), disease state (1–2), activities (0–3), metabolic demands (0–5) including Fever [0–3], cortisone [0–2], and degree of physical deficit (0–6) including loss of body fat or muscle mass [0–3], and edema or ascites [0–3], with higher scores indicating worse nutritional and health status (Table 2). Based on this scoring, the nutrition status of patients with cervical cancer is presented in Table 3. The nutritional risk is based on the total PG-SGA point score: none: 0–1, mild: 2–3, moderate: 4–8, and severe: ≥ 9. Category A: well-nourished score: 1–3, Category B: moderate risk of malnutrition: 4–8, and Category C: severe malnutrition with scores: 9+.

Food intake frequencies before and after diagnosis were recorded on a typical day before diagnosis and within 24 hours of the interview after the confirmed diagnosis, including the intake across the main food categories in Tanzania.

Blood nutritional biomarkers included inflammation indicators, such as white blood cells (WBC), indicators of neutropenia (neutrophil counts), red blood cells (RBC), and total number of platelets in the blood (PLT), indicators of inflammation and autoimmune diseases, such as the ratio of platelet and lymphocyte count (PLCR) and the ratio of platelet and neutrophil count (PLCC), heart and liver function indicators, such as hemoglobin (HGB), aspartate aminotransferase enzyme levels (AST), and alanine transaminase enzyme level (ALT, liver function indicator), bone and liver function indicator such as alkaline phosphatase enzyme levels (ALP), and kidney function health indicators, such as urea concentration in blood (UREA), and creatinine concentration in the blood (CRE-E). These blood markers were collected on-site from the patient’s medical records during management.

The questionnaires for nutritional assessment are included in the Supplementary materials.

### Covariate Assessment

Self-reported covariates included sociodemographic and lifestyle variables: age (years), marital status (married or not married), employment status (Yes/No), educational attainment (none, primary, secondary or above), number of people in the household, number of children, lack of housing security in the past six months (Yes/No), sold or thinking about selling property for treatment (Yes/No), lack of resources to buy medication (Yes/No), lack of food security (Yes/No), smoking status (former/never), alcohol use (current/former/never), physical activity (no/run/walk), transportation access (Yes/No), sexual activity (Yes/No), and HPV vaccine awareness (Yes/No). Clinical information included stage of cervical cancer (categorized as early-stage: I and II, and late-stage: III and IV), number of symptoms and comorbidities, edema (no, mild, moderate), weight change (no change/increase/decrease), change in food intake (no/normal food but < normal amount or eat less/ nutritional supplement or only liquids/ eat more), family history of cancer (Yes/No), physical function (mild/moderate/normal/severe), and body mass index (BMI, *kg/m*^*2*^, measured by the research assistant on-site).

## Statistical Analysis

All the analyses were performed using SAS (version 9.4), and results were reported at a 2-tailed α-level of 0.05. Descriptive analyses of nutritional status were performed using mean and standard deviation (SD) for nutritional assessment scores, mean blood biomarker levels, and dietary intake on a typical day before diagnosis and within 24 hours of interview after diagnosis. A comparison of the mean levels of these continuous factors by cancer stage was done using a t-test. A comparison between mean food intake frequencies before and after diagnosis was conducted using a paired t-test. Counts and percentages were calculated for categorical level patients’ characteristics, clinical information, lifestyle factors, and nutritional status outcomes such as global categorical rating and nutrition triage recommendation. A comparison of categorical level factors according to cancer stage was performed using the Chi-square test. Multiple imputation was used to impute the missing data in stage (n = 6), food intake (prior to and after diagnosis, n = 2), and blood nutritional markers (missing rate < 5%, n = 9).

Multivariable-adjusted generalized linear regression models were adopted to estimate the least square means and 95% confidence intervals (CIs) for nutrition assessment comparison between early and late cancer stages. Multivariable-adjusted logistic regression models were used to estimate the association between cancer stage and global category ratings with odds ratios and their 95% CIs. A backward stepwise selection procedure was adopted in the model to include sociodemographic and lifestyle covariates such as age, education, BMI (included in model 1), smoking, alcohol use, and physical activity (further included in model 2).

## Results

### Study population characteristics

Of the 184 patients, the majority were diagnosed with stage II and III cervical cancer (87.5%) and did not have a family history of cancer (81%) (Table 1). A higher proportion of patients were married, unemployed, had lower physical activity levels and educational attainment, and lacked reliable transportation and awareness of human papillomavirus (HPV) vaccination. Most of the patients did not actively smoke or drink alcohol. More than half of the patients had worsened physical function, and they did not engage in recent sexual activity. Patients experienced an average of 1.4 symptoms and comorbidities. Approximately 50% of patients consumed less food or relied on nutritional supplements or liquids (Table 3), and the majority of patients (58.7%) experienced weight loss (Table 1).

Tables 3 and 4 describe the patients’ overall nutritional status and dietary changes (before and after diagnosis). The mean nutritional risk score (1–24) was 6.5 ± 5.2 (median = 5) (Table 3). Based on the PG-GSA nutritional assessment score, 84.8% of patients are recommended for nutrition intervention to alleviate the risk of mild (27.7%), moderate (24.5%), or severe malnutrition (32.6%). 39.1% of the patients reported consuming everyday foods, but in smaller amounts than before the onset of cancer. A decrease in the mean daily intake before and after cancer diagnosis was observed in the intakes of cereals (*p* < 0.001), vegetables (dark leafy green vegetables (*p* = 0.04), orange vegetables (*p* < 0.001), and other vegetables (*p* < 0.001), and oils and fats (*p* < 0.001) (Table 4).

Table 5 shows the distribution of nutritional status by the early and late stages of cancer. A higher proportion of patients with moderate and severe risk of malnutrition was observed among those with late-stage disease (moderate malnutrition risk: late vs. early stage, 46.8% vs. 31.4%; risk of severe malnutrition: late vs. early, 31.9% vs. 16.8%; *p* = 0.001). Regarding the blood biomarkers, the higher mean levels of WBC (late vs. early: 9.0 ± 1.1 vs. 7.6 ± 0.6; *p* = 0.0216), PLT (late vs. early: 396.3 ± 39.4 vs. 347.5 ± 23.9; *p* = 0.0399), and PLCC (late vs. early: 101.7 ± 13.8 vs. 82.3 ± 1.6; *p* = 0.0294) can be observed in the patients with late-stage, as compared to the patients with early-stage, indicating a more severe inflammation level among patients with late-stage. The mean level of AST, a liver function test, was also higher in patients with late-stage (mean = 31.9 ± 5.9 for late-stage vs. mean = 24.5 ± 2.0 for early-stage; *p* = 0.0201). While similar patterns were also observed in other biomarkers such as ALT (*p* = 0.3839), ALP (*p* = 0.2910), Urea (*p* = 0.1832) and CRE-E (*p* = 0.123), these findings were not statistically significant.

Table 6 reveals the association between cervical cancer stage and nutritional status after adjusting for sociodemographic and lifestyle covariates. Patients with early-stage cervical cancer score 6.0 (95% CI 5.2, 6.9), while patients with late-stage cervical cancer have a score of 7.8 (95% CI 6.3, 9.2, p = 0.04). Further adjustment for other covariates, such as smoking, alcohol use or physical activity, did not have an additional effect.

## Discussion

To our knowledge, this is the first study to report on the nutritional status of patients with cervical cancer in Tanzania. The study revealed that the majority of patients with cervical cancer in this study had a low intake of food, including cereals, vegetables (such as orange vegetables, dark leafy green vegetables, and other vegetables), and oils and fats. The study also showed that a more advanced cancer stage was associated with a worse nutritional status. Finally, the study revealed that levels of inflammatory markers, such as WBC, PLCC, and heart and liver function markers, such as AST, were higher in patients with late-stage cervical cancer.

The decrease in food intake was a common observation in patients with cervical cancer in this study. Patients cannot maintain a good nutrition status when food intake is reduced to less than normal [16]. As such, malnutrition may likely occur in patients with cervical cancer. The reported significant reduction in the intake of certain foods rich in micronutrients, such as vegetables and cereals, can aggravate patient malnutrition, weakening patients’ overall health and diminishing their survival probability [17, 18]. Specifically, the reduced food groups, such as vegetables of all kinds, can be detrimental to cervical cancer patients’ health and survival outcomes. This is because consumption of vegetables and their included nutrients (such as vitamin C, vitamin E, carotene, carotenoid, zinc, iron, potassium, and antioxidants) may have protective effects against cervical cancer development, progression, and response to treatment [19–21].

Significant associations between late-stage cervical cancer and worsened nutritional status were found. By contrast, previous studies focused on malnutrition and survival outcomes. The scored PG-SGA is a well-documented tool to evaluate the nutritional status in patients with cervical or gynecological cancer [18, 22–25]. Studies have shown that PG-SGA could identify patients at nutritional risk ([25], and toxicity from chemotherapy is associated with malnutrition as identified by PG-GSA in patients with cervical cancer [22]. Several existing cohort studies reported malnutrition to be a predictor of a higher risk of premature death and lower cancer survival rates [17, 18]. The findings of the cohort studies are consistent with a retrospective cohort study, in which the poor nutritional status (reflected by the lower nutritional index assessed using blood nutritional biomarker and weight) predicted worsened survival rates and shortened survival of patients with advanced-stage cervical cancer [26]. Thus, there is a lack of research determining nutritional status based on the cervical cancer stage. The significant association between late-stage disease and the risk of severe malnutrition revealed in our study could help fill gaps in the existing literature, facilitating a deeper understanding of the determinants of malnourishment and their impact on patients’ survival and quality of life in developing countries. Also, the study findings showed the importance of providing nutritional support for patients with late-stage cervical cancer in developing countries.

Furthermore, higher mean levels of inflammatory markers (WBC, PLT) and liver and heart health markers (AST) were observed in patients with the late-stage disease. This finding was consistent with the literature that showed high levels of inflammatory markers in patients with late-stage cancer and can predict worsened survival outcomes [26]. Notably, the higher mean level of AST indicates that liver dysfunction may be unrelated to cervical cancer itself but to comorbidities common in late-stage patients.

The findings of the study highlight the importance of the provision of nutritional interventions, especially for late-stage cervical cancer, and also for other late-stage cancers. While cancer diagnosis and treatment have expanded in Tanzania [20, 21], efforts are needed to enhance patient care and provide nutritional and dietary support to augment treatment effectiveness.

This is the first study on nutrition and diet among cancer patients in Tanzania. This study utilized a dietary questionnaire of local food items and combined both dietary and laboratory markers for nutritional assessment. The study has limitations. Dietary information collected from self-reported FFQs through interviews may have included measurement errors [27], which might lead to null-toward biased results and underestimated postulated associations. Blood nutritional biomarkers were assessed at a single point in time, rather than through repeated measures during treatment. However, the nutritional status of patients is expected to worsen during the cancer treatment.

The high proportion of patients who lacked financial support for transportation to the cancer center shows the likelihood of patients’ sacrifice of their food intake for saving money for afford the transportation to the cancer treatment facility. The severity of the risk of malnutrition reported in this study was statistically associated with late-stage cervical cancer in Tanzania, suggesting that referral to nutritionists and nutrition intervention and support at hospital and community levels should be prioritized for the participants diagnosed with late-stage cervical cancer for better survival and quality of life in developing countries, like Tanzania.

## Supplementary Material

Supplementary Files

This is a list of supplementary files associated with this preprint. Click to download.

- DietarydiversitytoolquestionnaireTFNC.doc
- NutritionalAssessment.docx
- nutritionscreening.docx

## Figures and Tables

**Table 1:** Characteristics of patients with cervical cancer in Tanzania (N = 184).

Descriptive Information of Study Population	
**Patients’ Characteristics**	
Age, Mean ± S.D.	53.3 ± 11.1
Married (yes), n (%)	106 (57.6)
Unemployed (yes), n (%)	177 (96.2)
Education, %	
None	50 (27.2)
Primary	122 (66.3)
Secondary or above	12 (6.5)
**Patients’ Lifestyles**	
# People living in the household	4.9 ± 2.4
# Children	4.5 ± 2.2
Lack of housing security in the past 6 months (yes), n (%)	129 (70.1)
Sold or thinking about selling property to finance treatment (yes), n (%)	75 (40.8)
Lack of food security within the past 6 months (yes), n (%)	28 (15.2)
Smoking, n (%)	
Former smoker	3 (1.6)
Never smoker	181 (98.4)
Alcohol status, n (%)	
Current	4 (2.2)
Former	93 (50.5)
Never	87 (47.3)
Physical Activity, n (%)	
No	169 (91.9)
Run	5 (2.7)
Walk	10 (5.4)
Having transportation (yes), n (%)	80 (43.5)
Sexually active (yes), n (%)	54 (29.4)
Decided not to purchase medications for high expense within the past 6 months (yes),	53 (28.8)
n (%)	
Having HPV knowledge (yes), n (%)	62 (33.7)
**Patients’ Clinical Information**	
BMI, Mean ± S.D.	24.3 ± 5.4
Stage of cervical cancer, n (%)	
I	14 (7.6)
II	123 (66.9)
III	38 (20.6)
IV	9 (4.9)
Symptoms, Mean ± S.D.	1.4±1.8
Comorbidities, Mean ± S.D.	1.4± 0.5
Edema, n (%)	
No	178 (96.8)
Mild	3 (1.6)
Moderate	3 (1.6)
Weight change, n (%)	
No change	62 (33.7)
Increase	14 (7.6)
Decrease	108 (58.7)
Family history of cancer (yes), n (%)	35 (19.0)
Change in physical function, n (%)	
No change	85 (46.2)
Mild	67 (36.4)
Moderate	25 (13.6)
Severe	7 (3.8)

S.D: standard deviation

**Table 2:** Nutrition Assessment Score Categories

Item #	Category		Max-Score	Range
1	Weight	Decreased or not	1	0–1
2	Food Intake	Normal or decrease	4	0–4
3	Symptoms	No symptoms, NVCD*	3	0–3
4	Disease state	Cancer = 1, co-morbidity = 2	2	1–2
5	Activities	Active to bedridden (0–3)	3	0–3
6	Metabolic Demands	Fever (0–3), cortisone (0–2)	5	0–5
7	Physical Examination	loss of fat or muscle mass (0–3)	3	0–3
		edema or ascites (0–3)	3	0–3
Max. Score			**24**	**1–24**

* NVCD = Nausea, vomiting, constipation, or diarrhea

Metabolic demands include fever and cortisone medications

**Table 3:** Overall nutritional status of patients with cervical cancer included in the study

Overall Nutritional Status	
**Nutrition assessment score** (derived from the PG-SGA)	
Mean ± S.D.; Median (Range)	6.5 ± 5.2; 5.0 (1–24)
**Category rating**, n (%)	
A: well-nourished	81 (44.0)
B: moderate/suspected risk of malnutrition	65 (35.3)
C: Severe risk of malnutrition	38 (20.7)
**Recommendation** (nutritional intervention per total score), n (%)	
None (0–1)	28 (15.2)
Mild (2–3)	51 (27.7)
Moderate (4–8)	45 (24.5)
Severe (≥ 9)	60 (32.6)
**Change in food intake**, n (%)	
No change	93 (50.5)
Normal food but < normal amounts/eat less	72 (39.1)
Nutrition supplement/only liquids	15 (8.2)
Eat more	4 (2.2)

**Table 4:** Daily diet before and after diagnosis (N = 184)

Occurrence of dietary intake/day	Usual diet before diagnosis, Mean ± S.D.	Diet after diagnosis, Mean ± S.D.	Mean Difference	P value
Cereals	2.09 ± 0.40	0.98 ± 0.10	1.1 ±0.4	**< 0.001**
Orange vegetables	1.32 ± 0.50	0.98 ± 0.13	0.3 ± 0.5	**< 0.001**
White tubers	0.44 ± 0.51	0.44 ± 0.50	0.01 ±0.07	0.3186
Dark leafy green vegetables	0.96 ± 0.29	0.93 ± 0.25	0.02 ± 0.15	**0.0452**
Other vegetables	1.14± 0.42	0.97 ± 0.16	0.16 ± 0.4	**< 0.0001**
Vitamin A rich fruits	0.37 ± 0.49	0.37 ± 0.49	0±0.1	1.0
Other fruits	0.01 ±0.10	0.01 ±0.10	0±0	-
Organ meats	0.05 ± 0.22	0.04 ± 0.19	0.01 ± 0.1	0.1579
Flesh meats	0.57 ± 0.51	0.56 ± 0.50	0.01 ± 0.2	0.3186
Eggs	0.20 ± 0.40	0.20 ± 0.40	0 ± 0	-
Fish	0.60 ± 0.50	0.60 ± 0.49	−0.01 ± 0.2	0.1841
Legumes, nuts, and seeds	0.57 ± 0.56	0.54 ± 0.50	0.02 ± 0.2	0.1579
Dairy products	0.44 ± 0.53	0.42 ± 0.50	0.02 ± 0.2	0.258
Oils and fats	1.13± 0.46	0.97 ± 0.23	0.16 ± 0.38	**< 0.0001**
Sweets	0.90 ± 0.30	0.90 ± 0.31	0.01 ± 0.07	0.3186
Spices, condiments, and beverages	0.91 ±0.31	0.89 ± 0.31	0.02 ± 0.13	0.0833

For total score and blood biomarkers, t-test was used to examine the mean difference.

A chi-square test was performed to examine the proportion difference for category rating and triage recommendation.

**Table 5:** Nutritional status by cervical cancer stage (N = 184)

	Early stage (Stage I, II)	Late stage (Stage III, IV)	P value
Category rating, n (%)			**0.0011**
A: well-nourished	71 (51.8)	10 (21.3)	
B: moderate/suspected risk of malnutrition	43 (31.4)	22 (46.8)	
C: Severe risk of malnutrition	23 (16.8)	15 (31.9)	
Total	137 (100)	47 (100)	
Recommendation (nutritional intervention per total score), n (%)			**0.0061**
None (0–1)	45 (32.9)	6 (12.8)	
Mild (2–3)	28 (20.4)	17 (36.1)	
Moderate (4–8)	24 (17.5)	4 (8.5)	
Severe (≥ 9)	40 (29.2)	20 (42.6)	
Total	137 (100)	47 (100)	
Total Score (total count of nutritional indicators: 1–24), Mean ± S.D.	5.9 ± 0.8	8.0 ±1.4	**0.0173**
Blood Nutritional Biomarkers Mean ± S.D.			
White blood cells (WBC),10^9^/L	7.6 ± 0.6	9.0 ± 1.1	**0.0216**
Red blood cells (RBC),10^12^/L	4.2 ± 0.1	4.1 ±0.2	0.4841
Platelets (PLT), 10^9^/L	347.5 ± 23.9	396.3 ± 39.4	**0.0399**
Ratio of the platelet and lymphocyte count (PLCR), %	26.6 ± 1.5	25.7 ± 2.0	0.5631
Ratio of the platelet and neutrophil count (PLCC), %	82.3 ±1.6	101.7 ± 13.8	**0.0294**
Hemoglobin (HGB), g/dL	10.4 ±0.4	9.8 ± 0.8	0.1111
Alanine transaminase enzyme levels (ALT), U/L	18.7 ±2.7	21.3 ± 4.5	0.3839
Alkaline phosphatase enzyme levels (ALP), U/L	82.3 ± 1.6	101.7 ± 36.6	0.2910
Aspartate Aminotransferase enzyme levels (AST), U/L	24.5 ± 2.0	31.9 ± 5.9	**0.0201**
Urea concentration (UREA), mmol/L	4.2 ± 1.2	6.2 ± 2.8	0.1832
Creatinine (CRE-E), mmol/L	80.9 ± 6.3	93.3 ± 14.7	0.1230

For total score and blood biomarkers, t-test was used to examine the mean difference.

A chi-square test was performed to examine the proportion difference for category rating and triage recommendation.

**Table 6:** Prediction of the nutritional status by stage of cervical cancer diagnosis by consideration of the possible confounders. (N = 184)

Associations	Total score, least square mean (95% CI)	
	Model 1	Model 2
Early stage	6.0 (5.2, 6.9)	8.9 (6.3,11.4)
Late stage	7.8 (6.3, 9.2)	9.2 (6.5,11.9)
p-value	**0.04**	0.56

For the total score, a multivariable generalized linear regression model was used.

For category rating, multivariable logistic regression was used.

Compared to early-stage, it was less likely for a patient with late-stage cervical cancer to get a good category rating such as A or B.

Model 1: Adjusted for age, education, and BMI.

Model 2: Further adjusted for smoking, alcohol use, and physical activity.

## Data Availability

The datasets analyzed for this study can be found and obtained pending e-mail requests to the corresponding author.
